# Multi-Organ Contribution to the Metabolic Plasma Profile Using Hierarchical Modelling

**DOI:** 10.1371/journal.pone.0129260

**Published:** 2015-06-18

**Authors:** Frida Torell, Kate Bennett, Silvia Cereghini, Stefan Rännar, Katrin Lundstedt-Enkel, Thomas Moritz, Cecile Haumaitre, Johan Trygg, Torbjörn Lundstedt

**Affiliations:** 1 Computational Life Science Cluster (CLiC), Department of Chemistry, Umeå University, Umeå, Sweden; 2 Karlsruhe Institute of Technology, Karlsruhe, Germany; 3 AcureOmics AB, Umeå, Sweden; 4 CNRS, UMR7622, 75005, Paris, France; 5 Sorbonne Universités, UPMC, UMR7622, 75005, Paris, France; 6 Inserm U-1156, Paris, France; Instituto de Investigación Sanitaria INCLIVA, SPAIN

## Abstract

Hierarchical modelling was applied in order to identify the organs that contribute to the levels of metabolites in plasma. Plasma and organ samples from gut, kidney, liver, muscle and pancreas were obtained from mice. The samples were analysed using gas chromatography time-of-flight mass spectrometry (GC TOF-MS) at the Swedish Metabolomics centre, Umeå University, Sweden. The multivariate analysis was performed by means of principal component analysis (PCA) and orthogonal projections to latent structures (OPLS). The main goal of this study was to investigate how each organ contributes to the metabolic plasma profile. This was performed using hierarchical modelling. Each organ was found to have a unique metabolic profile. The hierarchical modelling showed that the gut, kidney and liver demonstrated the greatest contribution to the metabolic pattern of plasma. For example, we found that metabolites were absorbed in the gut and transported to the plasma. The kidneys excrete branched chain amino acids (BCAAs) and fatty acids are transported in the plasma to the muscles and liver. Lactic acid was also found to be transported from the pancreas to plasma. The results indicated that hierarchical modelling can be utilized to identify the organ contribution of unknown metabolites to the metabolic profile of plasma.

## Introduction

Chemical and biological systems are influenced by many interacting factors that must be considered simultaneously to optimize the understanding of the system in question [1]. Plasma or serum samples are the most commonly used samples types in metabolomic studies. In order to interpret and comprehend metabolomics data it is essential to determine the origin of the metabolites to increase the understanding of underlying biochemical pathways in disease and to assist in the design of individualized treatments and improved diagnosis. It is of great importance to understand disturbed biochemical pathways to target the correct organs with treatment. In this study, hierarchical modelling has been applied to identify the contribution that each organ has to the metabolic profile of plasma samples. Metabolic profiles were obtained for gut, kidney, liver, muscle, pancreas and plasma samples, using GC-TOF MS.

When applying PLS (partial least square projection to latent structures) and PCA modelling to complex data that includes multiple variables, the interpretation of the results becomes problematic. More sophisticated ways of analysing the data are required. In these situations it is advantageous to divide the variables into conceptually meaningful blocks and apply hierarchical modelling. The division into blocks results in two model levels. One level (the upper level) explains the relationship between each block whereas the other level (the lower level) contains detailed information regarding each individual block. [2] In this study, the upper level contained systemic variation from each compartment (e.g. systemic variation from the blocks representing the investigated organs and plasma) while the lower level contained information regarding their metabolic pattern.

The data that has been divided into blocks of logically related variables can be analysed by means of hierarchical principal component analysis (PCA) [3], partial least squares projection to latent structures (PLS) [4] and orthogonal-PLS (OPLS) [5] in addition to O2PLS [6]. By utilizing hierarchical modelling it is possible to penetrate complex data. [7] In the present study, OPLS was used to identify to what extent gut, kidney, liver, muscle and pancreas contribute to the metabolic pattern of plasma.

The application of hierarchical-principal component analysis allows visualization of multi-organ metabolic interactions. The metabolic interactions can be resolved at the compartment (the investigated organs and plasma) and pathway level. Therefore hierarchical modelling enables simultaneous modelling of the whole data set and simplifies interpretations [8]. Hierarchical modelling has been used in a variety of studies. These include studies regarding classification of G-protein-coupled receptors based on amino acid characterisation [9], transgenomic metabolic effects of probiotics [8], prediction of ligand binding to proteins [10], and in association studies of candidate genes and regions [11].

To the authors knowledge no multi-organ study has been performed that investigates how each organ contributes to the metabolic pattern that can be observed in plasma. The liver and kidneys play a key role in metabolism and the relative mass of muscle in a mammalian body is high. This may lead to the hypothesis that the metabolic pattern of plasma arises from the liver, kidneys and muscle. The main goal of this study was to address this issue by identifying the contribution of different organs to the metabolic profile of plasma, using hierarchical modelling [8, 9].

## Materials and Methods

### Materials

All standard reagents were of analytical grade or equivalent and obtained from Sigma-Aldrich (St Louis, MO, USA), Merck (Darmstadt, Germany) and J.T. Baker (Phillipsburg, NJ, USA). N-Methyl-N-trimethylsilyltrifluoroacetamide (MSTFA) plus 1% trimethylchlorosilane (TMCS) and pyridine were obtained from Thermo Fisher Scientific (Rockford, IL, USA). The stable isotope-labelled internal standards, [^13^C_5_]-proline, [^2^H_4_]-succinic acid, [^13^C_5_,^15^N]-glutamic acid, [1,2,3-^13^C_3_]-myristic acid, [^2^H_7_]-cholesterol and [^13^C_4_]-disodium α-ketoglutarate were purchased from Cambridge Isotope Laboratories (Andover, MA); [^13^C_6_]-glucose, [^13^C_12_]-sucrose, [^13^C_4_]-hexadecanoic acid and [^2^H_4_]-putrescine were purchased from Campro (Veenendaal, The Netherlands) and [^2^H_6_]-salicylic acid was obtained from Icon (Summit, NJ). Stock solutions of each internal standard were prepared in either Milli-Q water or methanol to a concentration of 0.5 μg/μL.

### Samples

Eight-month-old wild-type male mice of the mixed background 129sv x C57Bl/6N (n = 8) were individually placed in metabolic cages (Metabolic cage for mice, Tecniplast, UK), with access to food (ref A04-10 in powder, SAFE (Scientific animal food & engineering), France) and water. The animals were kept 5 days in the metabolic cages, 2 days as an adaptation period and 3 days of experiment. Each experimental day, animals were weighed. Food and water intake, urine volume and fecal weight for each mouse were recorded. Urine and feces were collected every 24 hours, and frozen at -80°C for storage until analysis. The third day of experiments and after 4h fasting, a blood sample from each animal was recovered by retro-orbital bleeding performed with a heparinized Pasteur pipette and collected in heparin tubes (Microvette CB300 LH, Sarstedt). Plasma was obtained from the blood sample by centrifugation (2000rpm, 5 min at 20°C), and frozen at -80°C. Animals were killed by cervical dislocation. Pancreas, liver, gut, kidney and muscle tissues were removed and dissected in cold HBSS solution (Hanks balanced salt solution, Life Technologies, US). Samples of each organ were washed in HBSS, collected in Cryo tubes, frozen in liquid nitrogen, and stored at -80°C until analysis. Animal experiments were conducted in accordance with French and European ethical legal guidelines and the local ethical committee for animal care (Comité d'éthique en expérimentation animale Charles Darwin N°5, approval number N° 01508.01) specifically approved this study.

### Metabolite extraction from tissues

Samples were ground to a fine powder under liquid nitrogen. A total of 1 ml methanol-chloroform-water (3:1:1), containing all eleven isotopically labelled internal standards (7 ng/μl) was added to 10 mg of frozen tissue. Samples were extracted using a MM 400 Vibration Mill (Retsch GmbH & Co. KG, Haan, Germany) at a frequency of 30 Hz for 3 min. A 3 mm tungsten carbide bead (Retsch GmbH & Co. KG, Haan, Germany) was added to each tube prior to mixing to increase the extraction efficiency. The beads were removed and the samples were centrifuged at 18 620 g for 15 min at 4°C. A volume of 200 μl supernatant was transferred to a GC vial and evaporated to dryness in a SpeedVac concentrator (Savant Instrument, Framingdale, NY, USA). All samples were stored at -80°C until analysis.

### Metabolite extraction from plasma

Frozen plasma was thawed at room temperature for 10 min and stored on ice (4°C). Plasma samples were prepared for GC-TOF-MS analysis by adding 900 μl extraction mix (methanol:H_2_O, 8:2, v/v), containing all eleven isotopically labelled internal standards (7 ng/μl), to 100 μl aliquots of plasma. Each sample was extracted vigorously using a MM 400 Vibration Mill (Retsch GmbH & Co. KG, Haan, Germany) at a frequency of 30 Hz for 3 min, followed by centrifugation at 18 620 g for 15 min at 4°C. A volume of 200 μl supernatant was transferred to a GC vial and evaporated to dryness in a SpeedVac concentrator (Savant Instrument, Framingdale, NY, USA).

### Derivatisation of samples

A 30 μl sample of methoxyamine (15 μg/μl) in pyridine was added to each GC vial and the resultant mixture was shaken vigorously for 10 min. Methoxymation was performed at room temperature for 16 h, followed by the addition of 30 μl MSTFA with 1% TMCS to each sample (brief vortex). Samples were left at room temperature for 1 h to allow silylation to occur, followed by the addition of 30 μl heptane (containing 15 ng/μl methyl stearate as an internal standard) and a brief vortex for 10 s. For selected samples technical replicates were also performed, where the whole extraction process was repeated (in triplicate) on the same plasma sample. For these triplicates, the mean values were used.

### GC-TOF-MS analysis

A volume of 1 μl of each derivatised sample was injected splitless by a CTC Combi Pal autosampler (CTC Analytics AG, Zwingen, Switzerland) into an Agilent 6980 GC equipped with a 10 m X 0.18 mm i.d. fused-silica capillary column chemically bonded with 0.18-um DB 5-MS stationary phase (J&W Scientific Folsom, CA). The injector temperature was set to 270°C. Helium was used as the carrier gas at a constant flow rate of 1 mL min^-1^ through the column. For every analysis, the purge time was set to 60 s at a purge flow rate of 20 mL min^-1^ and an equilibrium time of 1 min. The column temperature was held initially at 70°C for 2 min, then increased to 320°C at a rate of 30°C min^-1^, where it was held for 2 min. The column effluent was introduced into the ion source of a Pegasus III time-of-flight mass spectrometer (Leco Corp., St Joseph, MI). The ion source and transfer line temperatures were set to 200°C and 250°C, respectively. Ions were generated by a 70-ev electron beam at a current of 2.0 mA. Masses were acquired in the mass range 50–800 m/z at a rate of 30 spectra s^-1^. The acceleration voltage was turned on after a solvent delay of 150 s. The detector voltage was 1670 V.

### Data analysis of all samples analysed by GC/MS

Nonprocessed MS files from GC/TOFMS analysis were exported in NetCDF format to MATLAB software 7.11 (Mathworks, Natick, MA), where all data pretreatment procedures, such as baseline correction, chromatogram alignment, time-window setting and multivariate curve resolution (MCR) [12] were performed using custom scripts. Automatic peak detection and mass spectrum deconvolution with ChromaTof software were performed using a peak width set to 2 s.

For the identification of metabolites, NIST MS Search 2.0 software was used to compare the mass spectra of all detected compounds with spectra in the NIST library 2.0, the in-house mass spectra library established by Umeå Plant Science Centre and the mass spectra library maintained by the Max Planck Institute in Golm (http://csbdb.mpimp-golm.mpg.de/csbdb/gmd/gmd.html). A retention index comparison was performed, with a retention index deviation <+-10 (in addition to a high spectral match) resulting in a positive ID. LECO ChromaTOF software v4.32 (Leco Corp., St Joseph, MI) was also used as an additional tool for metabolite identification.

The data were normalised using all 11 internal standards (eluting over the whole chromatographic time range). To obtain accurate peak areas for the internal standards, 2 unique masses for each compound were specified and the samples were reprocessed using an in-house MATLAB 7.11 based script. A principal component analysis, using these peak areas, was calculated and the T-score value for each sample was used to normalise the resolved data by dividing the peak areas of each sample with the corresponding score value. Multivariate analysis was performed with SIMCA-P+ 12.0.1 software (Umetrics AB, Umeå, Sweden).

### Data analysis

The multivariate data analysis was performed using SIMCA-P+, version 13.0 (Umetrics AB, Umeå, Sweden). As a first step principal component analysis (PCA) [3] was applied, where the metabolite data from each organ (gut, kidney, liver, muscle and pancreas) was modelled together. PCA is an unsupervised multivariate technique where the variation in correlated variables are summarised into a smaller number of latent variables, so called principal components. The latent variables are used to describe the observations. Thereby the relationship between observations that are characterised by many variables can be visualised in low dimensional plots. [13]

**Fig 1:**
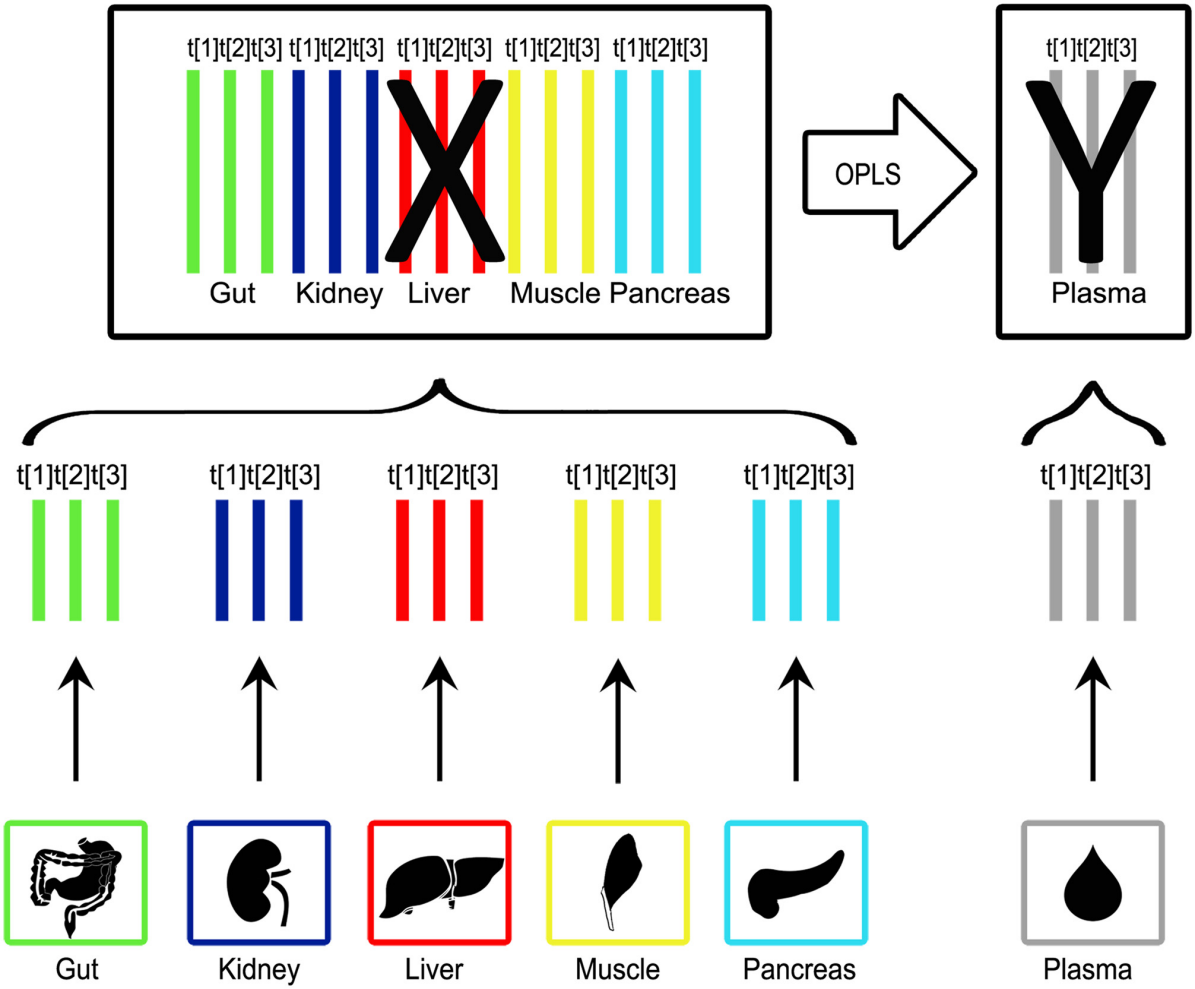
Overview of the pipeline for the hierarchical modelling. The normalised data from the GC-TOF MS analysis was used to create separate PCA models for each organ. The number of components for each PCA model was determined based on when the explained variation exceeded 50% to make the contribution comparable. The score vectors were collected from the PCA models and combined into a new dataset. In this data set the organ score vectors were concatenated into a score matrix (X) and the plasma score vectors were concatenated into a score matrix (Y). As a final step an OPLS model was calculated. This enabled the identification of the organs that gave a significant (by Jack-knifing) contribution to the metabolic profile of plasma.

Hierarchical modelling was thereafter applied to identify organs that contribute to the metabolic profile in plasma. One PCA model was calculated for each organ as well as plasma. Column centering and unit variance (UV) scaling was applied to the metabolite data, as pre-processing. The number of components for each PCA model was determined based on when the explained variation exceeded 50% to make the contribution comparable. The PCA model scores from each organ model were concatenated into a score matrix (X). Furthermore, the PCA model scores from the plasma model were concatenated into a score matrix (Y). In this way the variables were divided into conceptually meaningful blocks. The division into blocks result in two model levels. One level (the upper level) explains the relationship between blocks and the other level (the lower) contains detailed information about each block. [2] The upper level contained the systemic variation from each compartment (block) while the lower level contained information regarding their metabolic pattern. Orthogonal-PLS (OPLS) [5] was used to map the contribution of each organ to the overall metabolic profile found in plasma. To summarise the importance of the organs when it comes to explaining the metabolic profile of plasma a VIP (Variable Importance in the Projection) plot was created. Whether a block had a significant contribution was determined by consulting the VIP plot. The VIP plot is a way to visualise the importance of the variables both to explain X (in this case organ) and to correlate to Y (in this case plasma). The VIP value for a variable is calculated by weighing the squared OPLS loading weights against the explained sum of squares for each model component. [14] In the upper level the loading plot was used to identify the direction of the influence of the different score component from the various organs. In the lower level the loadings were used to uncover variables contributing to the hierarchical OPLS super scores. The outline of the hierarchical modelling was obtained (Fig 1).

## Results

### Distribution of metabolites in different organs

Metabolic profiling was performed on five different mouse organs (liver, kidney, gut, pancreas and muscle) using GCMS. The metabolomics data was used to investigate the distribution of the metabolites in the different organs. There was a great overlap in the identification of the metabolites in each organ. Only a few exceptions were found, for example, cholic acid was only identified in the gut and fatty acid methyl esters (methyl hexadecanoate, methyl linolate, (9)-Z-octadecenoic acid methyl ester) were only identified in the pancreas. In addition, creatinine was only found in gut, muscle and pancreas, where a great majority was found in the muscle.

### Relationship of organs to the metabolic profile of plasma

**Fig 2:**
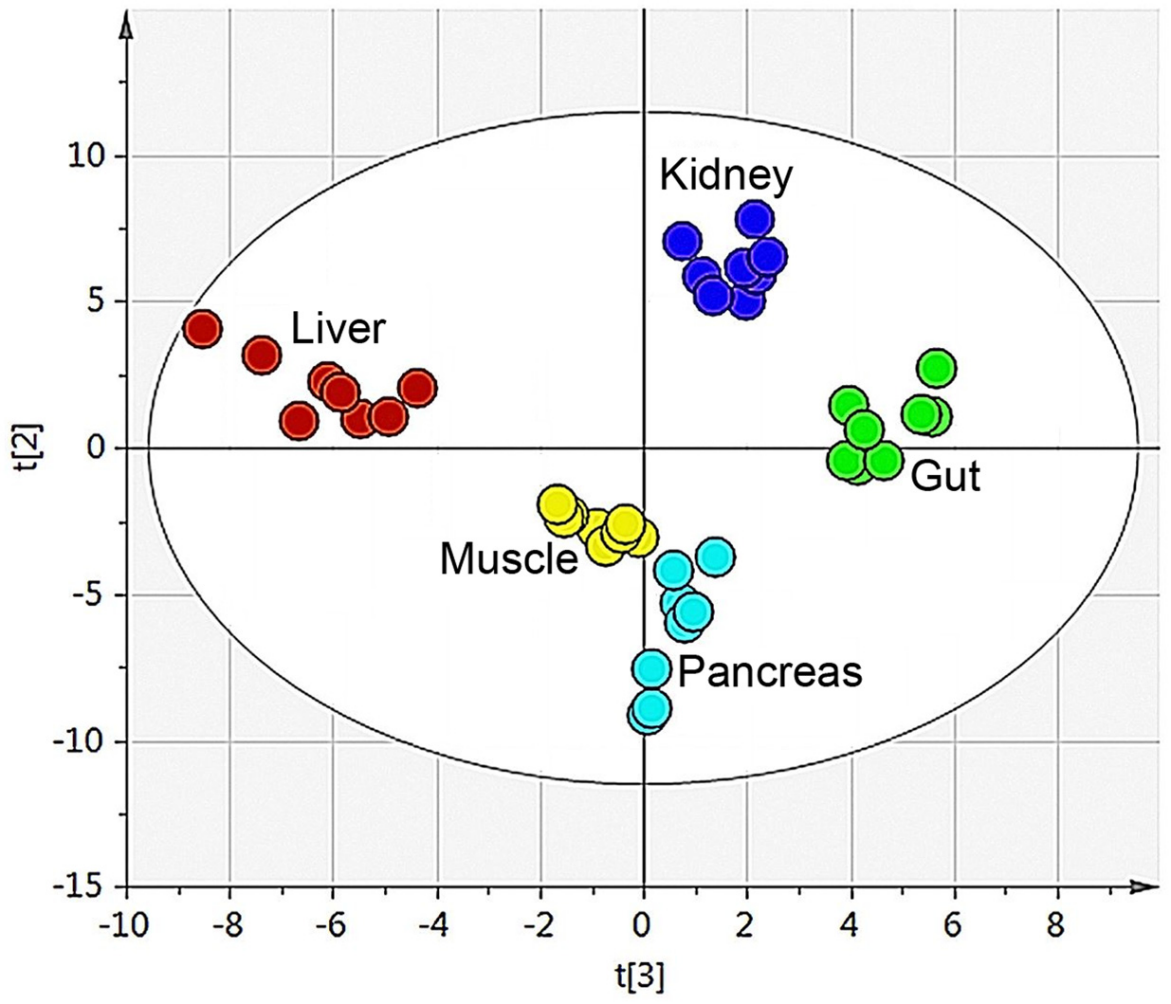
Organ sample PCA score plot. The score plot for the four component PCA model (R^2^X(cum) = 0.67 and Q^2^(cum) = 0.52) for the five different organs (gut, kidney, liver, muscle and pancreas). The second and third component best described the organs and explained 30% of the variation.

**Fig 3:**
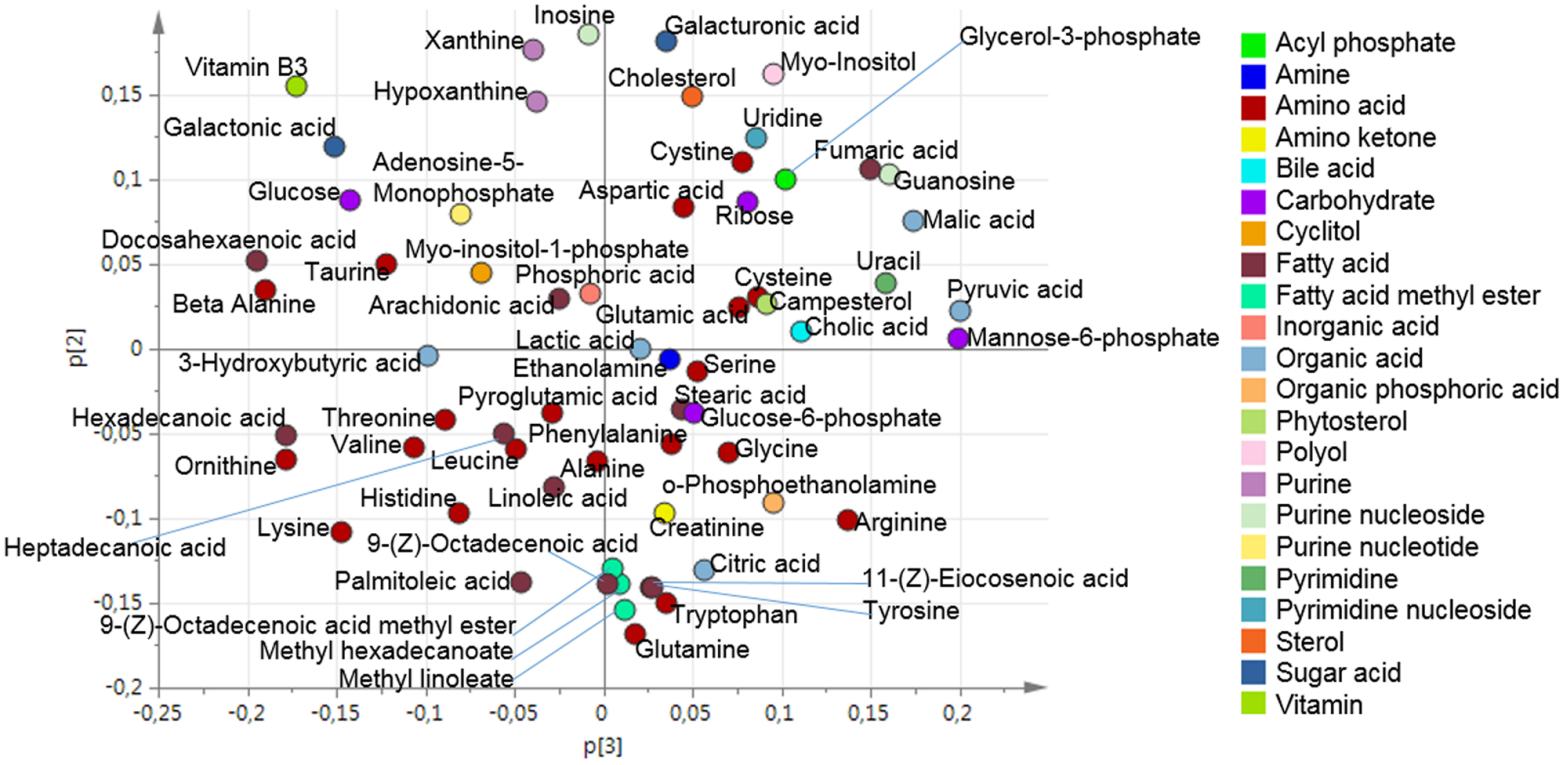
Organ sample PCA loading plot. The loading plot for the four component PCA model (R^2^X(cum) = 0.67 and Q^2^(cum) = 0.52) for the five different organs (gut, kidney, liver, muscle and pancreas).

All putative metabolites were included in the multivariate analysis of the data. By means of principal component analysis (PCA) an overview was obtained of the combined organ data to identify and visualise any groupings, trends and outliers [3]. The technical replicates that were used (triplicates of each sample) were all found in the same quadrant in the score plot for each separate sample. This indicated the high reproducibility of the analytical method. A mean value of the triplicates was calculated and a new PCA was performed. The PCA resulted in a four component model (R^2^X(cum) = 0.67 and Q^2^(cum) = 0.52). The second and third component best described the separation between the organs, Figs 2 and 3. The first component did not show any clear separation between the organs, see S1 Fig

### Hierarchical modelling

The initial step of the hierarchical modelling was to calculate separate, UV-scaled, PCA models for each organ. The number of components for each PCA was determined based on when the explained variation exceeded 50%. This was performed to make the contribution comparable. The GCMS data for the organ samples were analysed simultaneously. Only the metabolites common to each organ were used in the hierarchical modelling. Based on this, three components were selected for each organ and is shown in Fig 1. The explained variation exceeded 60% for all of the organs, including plasma. The calculated, three component PCA models are summarised in Table 1.

**Table 1:** Summary of organ PCA models used in hierarchical modelling.

Compartment	Type	A	N	R^2^X(cum)
Gut	PCA-X	3	8	0.73
Kidney	PCA-X	3	8	0.66
Liver	PCA-X	3	8	0.60
Muscle	PCA-X	3	8	0.64
Pancreas	PCA-X	3	8	0.66
Plasma	PCA-X	3	8	0.65

The PCA models that were used in the hierarchical modelling are listed here. The number of components used for each compartment is listed under column A. N represents the number of samples that the PCA model is based on. The variation explained by each model is listed under R^2^X.

**Fig 4:**
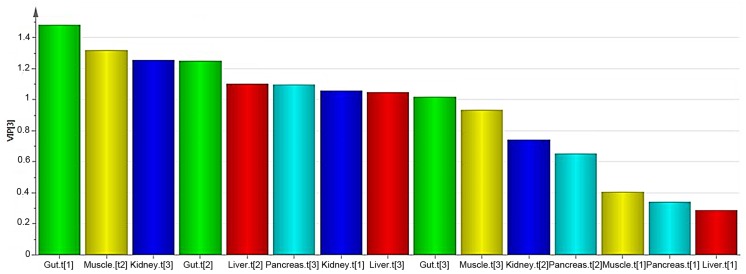
VIP plot visualising organ contribution. The VIP plot shows the importance of each variable when explaining X and the correlation to Y. The importance of the variable is indicated by the height of the bar.

The three score vectors from the PCA model of each organ were concatenated into a score matrix (X), while the three score vectors from the plasma were concatenated into a score matrix (Y). Thereby, the metabolomic data was divided into meaningful blocks. In the resulting hierarchical model the upper level explained the relationship between organs and plasma and, the lower level contained detailed information regarding each of the investigated organs and plasma. OPLS [5] was used to map the contribution of each organ to the overall metabolic profile found in plasma. An OPLS model separated the plasma related (predictive) variation from the variation uncorrelated to plasma (orthogonal variation). UV scaling was applied prior to PCA modelling of each organ. The OPLS model resulted in a 3+1+0 component model where R^2^X(cum) = 0.64, R^2^Y(cum) = 0.98 and Q^2^(cum) = 0.47. The VIP plot was used to visualise the dynamics of the metabolic pattern in plasma (Fig 4). A VIP value over one indicated that the variable was important when explaining X and it’s correlation to Y.

Combining the R^2^X(cum) values with the VIP values for the respective components facilitated the interpretation of the contribution of the components to the metabolic profile of plasma. In this case, R^2^X(cum) showed how much of the variation of the respective organ was explained by a certain component. The VIP value showed how important each of the components were when explaining the contributions to the plasma metabolite levels. A summary of the R^2^X(cum) and VIP values can be found in Table 2.

**Table 2:** Summary of R^2^X(cum) and VIP.

Compartment	Component	R^2^X(cum)	VIP value
Gut	Gut.t[1]	0.41	1.48
	Gut.t[2]	0.18	1.25
	Gut.t[3]	0.14	1.02
Kidney	Kidney.t[1]	0.30	1.06
	Kidney.t[2]	0.20	0.75
	Kidney.t[3]	0.16	1.26
Liver	Liver.t[1]	0.23	0.29
	Liver.t[2]	0.20	1.10
	Liver.t[3]	0.17	1.05
Muscle	Muscle.t[1]	0.26	0.41
	Muscle.t[2]	0.23	1.32
	Muscle.t[3]	0.15	0.94
Pancreas	Pancreas.t[1]	0.32	0.34
	Pancreas.t[2]	0.17	0.66
	Pancreas.t[3]	0.17	1.10

The R^2^X(cum) and VIP values for each of the components. R^2^X(cum) showed how much of the metabolic variation that each component explained. The VIP value indicated the importance of each of the components. VIP values over one indicated that the component was important when it came to explaining the contributions of each organ to the metabolite levels of plasma.

**Fig 5:**
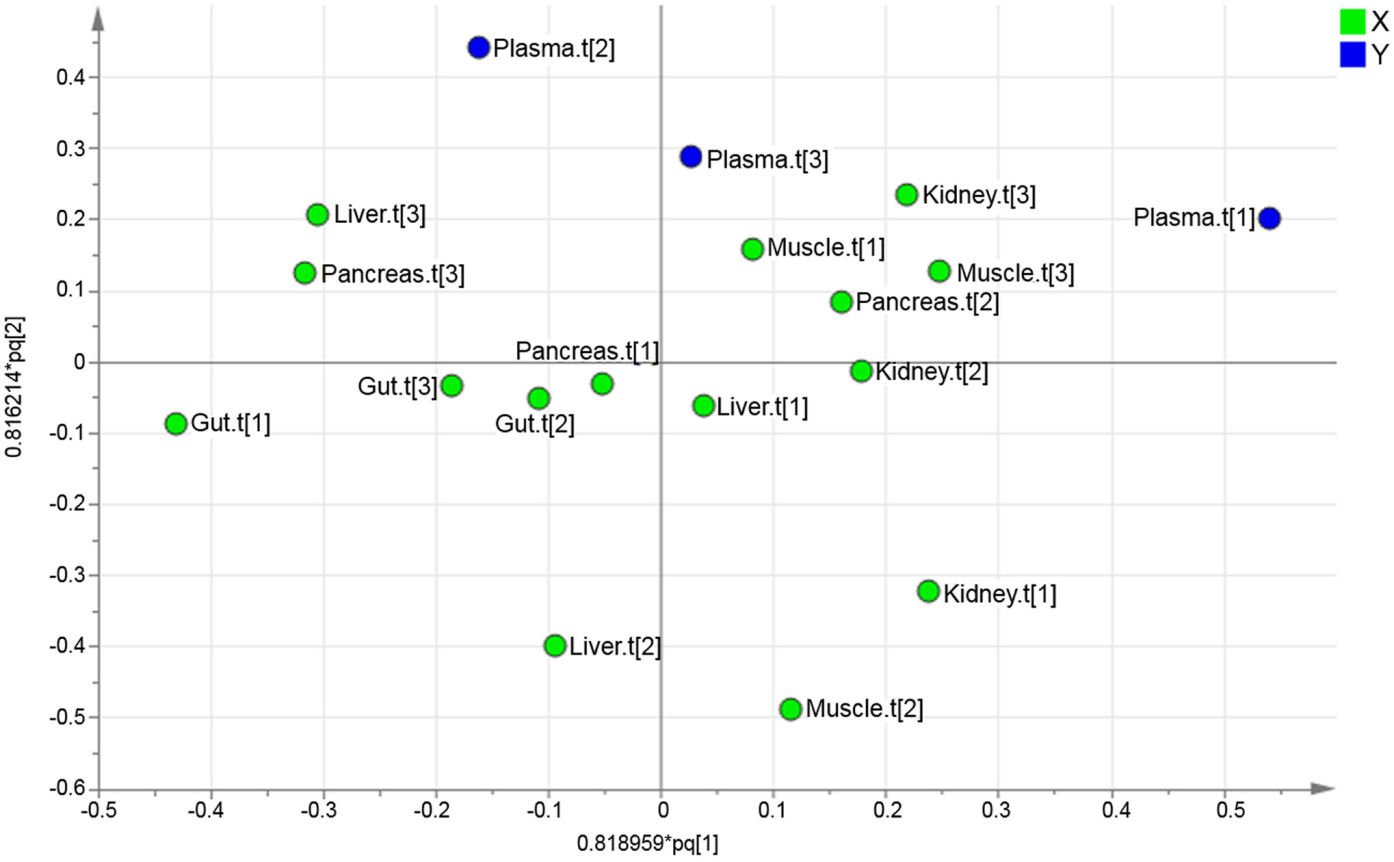
Hierarchical OPLS loading plot. The upper level loading plot can be used to identify the extent to which the different organ components influence the plasma components.

The upper level loading plot was used to find the direction of the contribution of each of the investigated organs to the metabolic profile of plasma (Fig 5).

**Fig 6:**
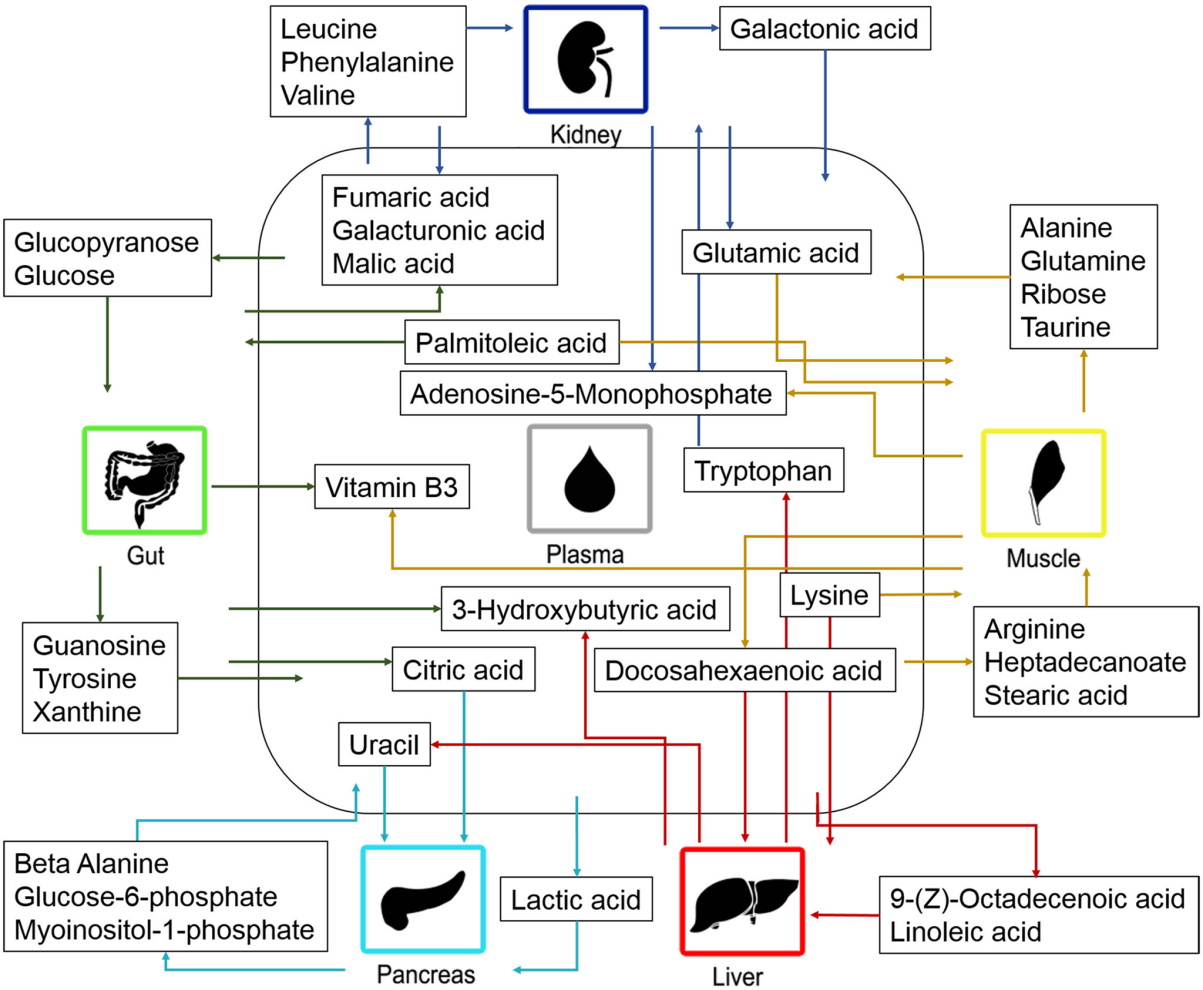
Metabolite contribution by the organs to plasma. The contribution of metabolites between plasma and the investigated organs. The arrows indicate the direction of the metabolic contribution.

To uncover variables contributing to the OPLS super scores, the loadings at the base level PCA were further investigated. The score vectors summarize the systemic variation from each compartment/organ. The lower level loading plots contain information regarding which of the original variables that were important. Therefore, the loading plots were used to identify how the organs contribute with metabolites to the plasma. Initially, the upper level loading plot was investigated. By identifying the directions in the loading plot it was found that Gut.t[1] had the greatest negative contribution to Plasma.t[1]. In contrast, Muscle.t[3] and Kidney.t[3] had the greatest positive contribution to Plasma.t[1]. Kidney.t[1] and Muslce.t[2] had the greatest negative contribution to Plasma.t[2]. Liver.t[2] had the greatest negative contribution to Plasma.t[3]. The contribution that each organ had to the metabolic profile of plasma was identified by applying the direction, from the upper level loading, onto the lower level loadings. Thereby, the metabolite contribution from the investigated organs to plasma could be found. The contribution of the investigated organs to the metabolic pattern found in plasma is summarized in Fig 6.

## Discussion

### Distribution of metabolites in different organs

The major goal of this study was to verify that hierarchical modelling can be used to identify the extent of metabolite contribution to what is observed in plasma. The initial step was to validate the analytical method by looking at metabolite distribution in the various tissue samples. The findings were compared to what is previously known on the location of metabolites. The metabolites were common to all tissue samples with a few exceptions. The investigation into the distribution of the identified metabolites showed that the gut contained the highest levels of the bile acid, cholic acid. This could have been explained by the fact that the gall bladder releases cholic acid into the gut in order to enable absorption of triglycerides, cholesterol, and lipid-soluble vitamins [15,16]. Creatinine was found in higher levels in the muscle samples. Creatinine is a breakdown product of creatine in the muscles, which corresponds well to our finding [17]. Finally, fatty acid methyl esters (methyl hexadecanoate, methyl linoleate, (9Z)-octadecenoic acid methyl ester) were present in much higher levels in the pancreas. This indicated a higher rate of biosynthesis of fatty acid methyl esters in pancreas or an accumulation of them there. It has previously been suggested that fatty acid methyl esters accumulate in the pancreas, which corresponds well with this study [18].

### Relationship of organs to the metabolic profile of plasma

The second step was to look at the distribution of the organ samples in the PCA. The score plot from the PCA model for the five different organs (gut, kidney, liver, muscle and pancreas) showed clear groupings and a great separation between the different tissue types. Each organ had a distinctive metabolic profile arising from the unique metabolic functions that each organ had, the different nutrients utilised in addition to the compounds produced and/or stored in the organ [19].

### Hierarchical modelling

The third step of the study was to identify the extent, to which each of the investigated organs contribute to the metabolic profile of plasma. The analysis of linkage between metabolic organ profiles and plasma profiles revealed that all of the investigated organs contribute to the metabolic pattern of plasma. The main contributor to plasma was the gut. In general, nutrients are absorbed in the intestine and transported by the cardiovascular system to all body cells, whereas the liver plays an important role in digestion and the kidneys are crucial for the excretion of metabolites [20]. This may explain why the kidney and liver were found to be the second and third greatest contributor, respectively. The liver and muscle were found to contribute to the plasma metabolite levels to an approximately equal extent. Out of the investigated organs, pancreas was found to have the smallest contribution to the levels of plasma metabolites.

Organ contribution to the metabolite levels observed in plasma involves highly active and regulated processes that provide metabolites to all tissues of the body, to be utilized in many processes, for example, protein synthesis and energy metabolism. The distribution of different classes of metabolites, including fatty acids, amino acids, sugars and TCA cycle intermediates, was investigated in all organs in relation to plasma. It was important to show that the findings corresponded well to previous knowledge of metabolism to confirm the validity of our method.

#### Fatty acids

It is well known that free fatty acids and mono-glycerides are absorbed by the intestine, triglycerides are resynthesized in the intestinal lumen and the fatty acids are transported to the lymphatic capillary in the form of chylomicron. Finally, the chylomicrons are transported from the lymph to the blood. [21] This could be the reason why the gut did not contribute with free fatty acids to the plasma. In the present study, palmitoleic acid was found to be absorbed by the gut and may be explained by fatty acids, e.g. palmitoleic acid, which work as signalling molecules for protein secretion from the intestine [22].

We also found the transportation of fatty acids to the liver (docosahexaenoic acid, linoleic acid and 9-(Z)-octadecenoic acid) and muscles (heptadecanoic acid, palmitoleic acid and stearic acid). The reason for this may have been that fatty acids undergo beta-oxidation in the liver and muscles. Genes for fatty acid oxidation have also been found to be expressed in liver, kidney and muscle [23]. Fatty acids derived from the diet or synthesized by the liver are esterified in the liver and secreted into the blood stream as very low density lipoprotein (VLDL). This may explain why no fatty acids were found to be transported from the liver to plasma, in our study. The muscles utilize fatty acids, glucose and ketone bodies as the major source of energy [19]. This could explain why the major proportion of fatty acids is transported to the muscles. When the levels of triaglycerols are lowered, long-chain n-3 fatty acids are released by the muscles [24]. This corresponds well with the observation that the n-3 fatty acids, docosahexaenoic acid, were found to be released by the muscles.

#### Amino acids

A wide range of amino acids were identified in the organs and plasma samples. The vast majority of the findings regarding the distribution of amino acids corresponded well with previous knowledge on amino acid metabolism. For example, we observed transport of amino acids that had been absorbed from the small intestine and transported to the liver where they are known to be catabolized [25–26]. An exception to this rule is tryptophan. In the present study, the liver was found to release tryptophan into the plasma, which corresponded well with what is known previously. Dietary tryptophan is also transported to the liver, where it is used for e.g. protein synthesis. The remaining amounts of tryptophan are released by the liver to be used elsewhere in the body. [27].

Amino acids are transported in and out of muscles utilizing a variety of amino acid transporters. [28] In the present study, arginine, glutamic acid and lysine, were found to be absorbed by the muscles. This corresponds well with previous studies where these amino acids are involved in different intracellular processes [29]. We observed that glutamine and alanine were released by the muscles. The muscles release nitrogen in the form of alanine since the muscle cannot produce urea. [19] It has been well documented that glutamine and alanine account for approximately 50% of the amino acid released by skeletal muscles [30]. In addition, we found that taurine is transported from the muscles to the plasma. It has been shown that taurine is important for proper maintenance and function of skeletal muscles [31]. In addition, exercising has been associated with decreased levels of taurine in the muscles and increased levels of taurine in the plasma, which may help explain our finding if the mice had been active prior to investigation [32, 33].

In the present study, there are signs that the kidneys are excreting aromatic amino acids (phenylalanine and tryptophan) and branched chain amino acids (leucine and valine) while the acidic amino acid, glutamic acid, were found to be transported from the kidneys to the plasma. Renal amino acid metabolism plays a key role in acid-base balance regulation via glutamine hydrolysis and ammonia excretion [34]. The body is not capable of storing amino acids. Therefore excess amino acids e.g. excess branched chain amino acids are rapidly catabolized and excreted via the kidneys, which corresponds well to our finding [30, 35]. Renal phenylalanine absorption and excretion have previously been observed in other studies, while findings regarding tryptophan have been inconclusive [35]. Our findings suggest that both the branched chain amino acids and the aromatic amino acids are excreted by the kidneys. The finding that acidic amino acids (glutamic acid, aspartic acid) are highly reabsorbed from the kidney to the plasma, is in line with previous knowledge [36–38].

#### Sugars

It is well known that sugars are ingested in the diet or are produced by gluconeogenesis in the liver or kidney and transported to other tissues via the bloodstream [39]. In the present study, galacturonic acid was found to be absorbed by the intestine and transported to the blood, which is in line with previous knowledge [40]. Glucose and glucopyranose are rapidly absorbed by the intestine and transported to the bloodstream [39]. In contrast, we found glucose to be transported to the gut. It should be considered that the mice had been fasting, for four hours, prior to collection of the organs. The absorption of sugars from the intestine is rapid. In parallel, glucose is required in every cell in the body for energy, including the cells of the gut, which may have explained transport into the gut during the fasting period.

In the present study, glucose-6-phosphate and myoinositol 1-phosphate were found to be released by the pancreas. Glucose is transported to and activated in the liver and/or pancreas by the formation of glucose 6-phosphate [41]. The transportation of these glucose-6-phosphate and myoinositol-1-phosphate between the pancreas and plasma has not been fully explored, but here the metabolites were found to be transported from the pancreas to the plasma. Myoinositol 1-phosphate synthetase is the enzyme that interconverts glucose 6-phosphate and myoinositol-1-phosphate and is highly expressed in the pancreas [42]. This indicates that this enzyme may also function in the pancreas to produce these metabolites, which can then be resale to the plasma.

In the present study, the muscles were found to contribute to the levels of ribose found in plasma. Ribose can be synthesized in skeletal muscles [43]. This could be the explanation for the release of ribose from the muscles.

In the present study, galacturoinc acid and galactonic acid was found to be transported from the kidneys to the plasma. It is well know that sugar acids are reabsorbed from the kidneys to the plasma [44].

#### TCA cycle intermediates

Citric acid, fumaric acid and malic acid were all found to be transported from the gut to the plasma. This corresponds well to previous studies that showed TCA cycle intermediates to be absorbed in the intestine and transported into the blood stream [45–47]. Fumaric acid and malic acid were also found to be transported from the kidney to the plasma. A high rate of reabsorption from the kidneys to the blood has also been observed previously [48].

The transportation of citric acid and lactic acid from plasma to the pancreas has not been fully explored and understood. However, we showed that both citric acid and lactic acid were transported from the plasma to the pancreas. Coincidentally, citric acid has been found to be involved in insulin secretion in the beta cells in the pancreas [49]. Moreover, lactic acid is transported over plasma membranes, including those in the pancreas, utilizing proton-linked monocarboxylate transporters [50].

#### Ketone bodies

Ketones bodies are produced as a result of fatty acid catabolism in the liver and are transported by the blood to other tissues where they are oxidized in the TCA cycle [21]. In the present study, the ketone body, 3-hydroxybutyric acid, was found to be absorbed by the intestine and transported into the blood stream, which corresponds well to previous knowledge [51]. In addition, 3-hydroxybutyric acid was found to be transported from the liver to the plasma. This may be explained by synthesis of 3-hydroxybutyric acid in the liver from acetyl-CoA [52, 21].

#### Nucleotides

Nucleotides are important in the cellular metabolism and essential in cellular energy transactions [53]. Our findings regarding purines show that guanosine and xanthine are transported from the gut to plasma. This is in line with the fact that purines are absorbed by the intestine and transported to the blood using an active transport mechanisms [54].

In this study, the liver was found to contribute to the level of uracil in plasma. Uracil can be synthesized in the mouse liver. Therefore, increased synthesis of uracil, in the mouse liver, results in increased levels of uracil in the blood. [55] In the present study, uracil was transported into the pancreas while beta-alanine was transported out of the pancreas. The breakdown product of uracil is beta-alanine [56]. Enzymes responsible for the breakdown of uracil to beta-alanine are expressed in pancreas, among other tissues [57]. The transportation of uracil and beta-alanine in pancreas has not been fully investigated. However, our results suggest that that uracil is converted to beta-alanine in the pancreas and is released into the blood stream as beta-alanine.

In the present study, both the muscles and kidneys were found to contribute to the plasma level of adenosine monophosphate (AMP). The muscles utilize adenosine triphosphate (ATP) to produce energy and as a result AMP is produced. AMP is a metabolite that inhibits energy production in the muscles and is rapidly broken down in this tissue [39]. In addition, glumural filtration involves dephosphorylation of ATP to AMP [58, 59]. In the kidneys, AMP is converted to cyclic AMP, inosine, adenosine and guanosine. [59] The extent to which the muscles and kidneys contribute to the levels of AMP in the plasma has not been fully investigated. However, our findings suggests that a proportion of the AMP that is produced in these organs reaches the blood stream.

#### Vitamins

In the present study both gut and muscle were found to contribute to the levels of vitamin B3 found in plasma. Vitamin B3 (Niacin) is absorbed in the intestine and transported into the blood stream, which corresponds well with our finding [60]. In animals, vitamin B3 is stored in the liver, muscle and heart [61]. Thereby, the direction of the metabolite contribution of vitamin B3, in mouse muscle, will vary. The vitamin will be absorbed from plasma, to be stored in the muscles, but can also be released from the muscle tissue when required.

The metabolic flow that was identified utilizing hierarchical modelling corresponded well with previous knowledge on metabolism, which confirmed the validity of our method. The analysis also provided new insights into the contribution of different organs to the metabolites observed in plasma. However, it is important to consider that metabolic processes are highly active and dynamic. Many metabolites will be transported both to and from different organs, depending on where the metabolites are needed. It must be noted that, in this study, we were only able to investigate a snapshot of metabolic activity. The metabolic flux of metabolites is not static but a dynamic process where metabolites can be transported across membranes to be utilized where they are required. By only obtaining a snapshot of activity it is not possible to observe the dynamics of the metabolic flow between organs. However, the small sample mass that is required (10 mg) for the analysis opens up the possible use of biopsies. There is therefore the potential to use time-series to obtain a more dynamic picture. The use of hierarchical modelling in the study of organ contribution to the metabolic pattern of plasma has proven to be a useful tool, i.e. may provide information regarding disturbed biochemical pathways in disease.

## Conclusion

Hierarchical modelling can be used to investigate to what extent the different organs contribute to the levels of metabolites observed in plasma. In the present study, it was found that the combined influence of the gut displayed the greatest contribution. In addition, kidney was found to have the second highest influence. Furthermore, muscle and liver were found to contribute to an approximately equal extent to the metabolic pattern found in plasma. Finally, the pancreas was found to have the smallest contribution, out of all the investigated organs.

We have also shown that it is possible to determine the level of organ contribution, of both known and unknown metabolites, to plasma by utilizing hierarchical modelling. The metabolomic analysis of both biofluids and tissue will provide a comprehensive picture of the metabolome. It is of great importance to understand the distribution of the metabolites in both organs and plasma in order to determine disturbed biochemical pathways in disease. This may provide important insights into the pathogenesis of disease and in the discovery of potential targets for treatment. Additional investigations should be performed in the field of metabolomics to explore inter-organ and inter-tissue transport in multiorgan systems. What is proposed here is a novel approach to study the organ contribution to plasma. The combination of tissue and biofluid metabolomics may prove a powerful tool in the discovery of potential targets for treatment and monitoring of disease.

## Supporting Information

S1 Fig First component score column plot. The first component does not separate the organs samples. The second and third component better described the separation between the organs. The first component explained 27% of the variation.
(TIF)
