# Supplementary figures and images for: Multi-Organ Contribution to the Metabolic Plasma Profile Using Hierarchical Modelling
Source: PLoS One. 2015 Jun 18;10(6):e0129260. doi: 10.1371/journal.pone.0129260 (PMC4472231; doi:10.1371/journal.pone.0129260)

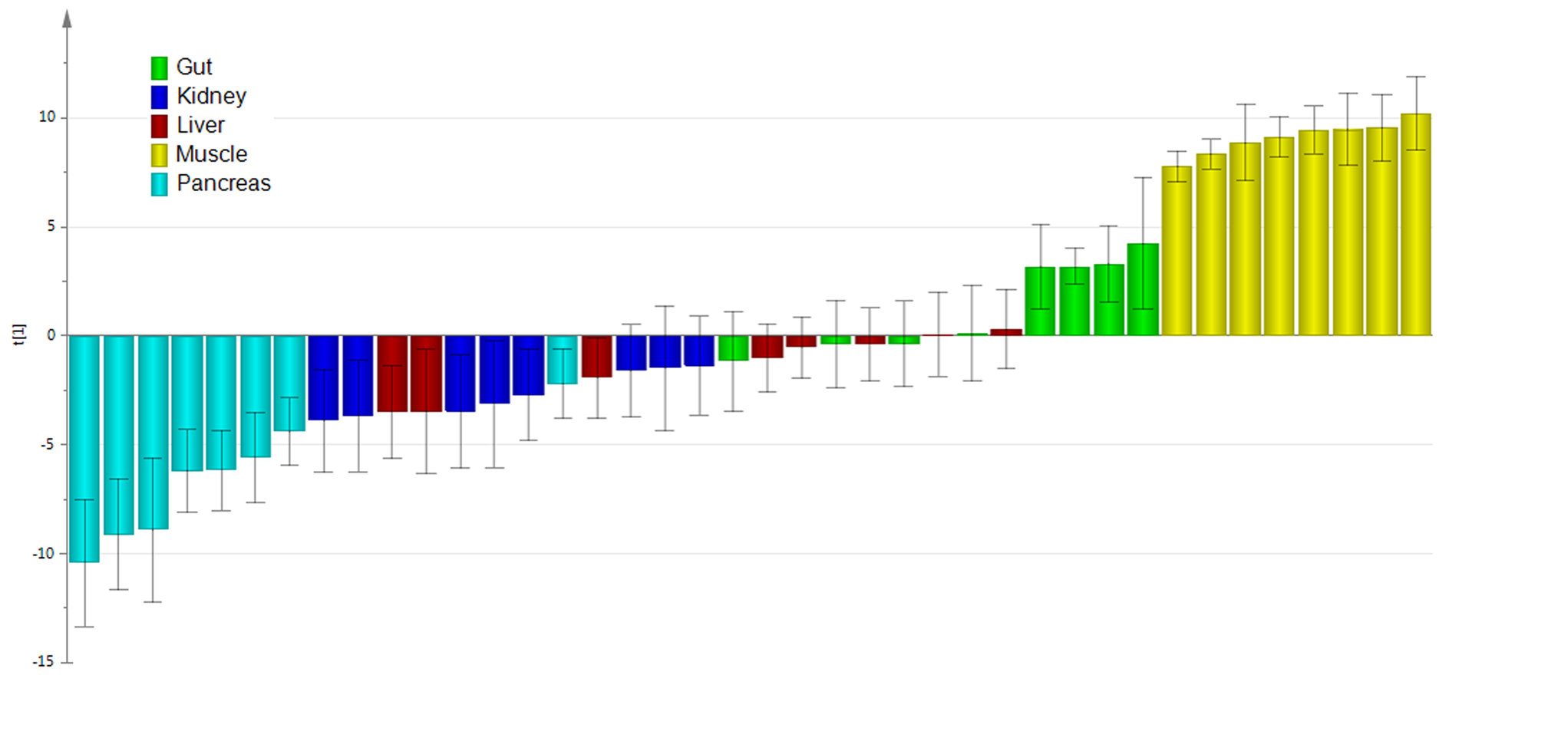

Supplement: S1 Fig — The first component does not separate the organs samples. The second and third component better described the separation between the organs. The first component explained 27% of the variation. (TIF) [file pone.0129260.s001.tif]
